# Laryngeal Sarcomatoid Carcinoma With Rhabdomyoblastic Differentiation: A Potential Pitfall for Misdiagnosis As Rhabdomyosarcoma

**DOI:** 10.7759/cureus.40990

**Published:** 2023-06-26

**Authors:** Billie Shine, Juan Carlos Alvarez Moreno, Orly Coblens, Suimin Qiu, Cecilia G Clement

**Affiliations:** 1 Pathology, University of Texas Medical Branch, Galveston, USA; 2 Otolaryngology, University of Texas Medical Branch, Galveston, USA

**Keywords:** rhabdomyoblastic differentiation, rhabdomyosarcoma, spindle cell squamous carcinoma, sarcomatoid carcinoma, larynx

## Abstract

Sarcomatoid carcinoma (SC) of the larynx is an uncommon subtype of squamous cell carcinoma which shows both squamous carcinoma and a sarcomatous component, including heterologous elements. The presence of rhabdomyosarcomatous elements in the larynx is extremely rare. Diagnosis of SC can be particularly challenging when the malignant epithelial component is not evident. We present a case of SC in a 72-year-old man with a superficial exophytic mass in the vocal cord initially misdiagnosed as rhabdomyosarcoma due to a predominant spindle cell component with rhabdomyoblastic features by morphology and immunohistochemistry. This case report aims to increase awareness that a rhabdomyoblastic heterologous component can be present in SC of the larynx and to consider this diagnosis in a mucosal exophytic malignant spindle cell neoplasm, even in the absence of epithelial differentiation.

## Introduction

Sarcomatoid carcinoma (SC), also known as spindle cell squamous carcinoma, is an uncommon subtype of squamous cell carcinoma composed of a malignant spindle and/or epithelioid pleomorphic cell component associated with intraepithelial dysplasia and/or invasive squamous cell carcinoma [1]. The sarcomatous component can show heterologous elements, including osteo-chondrosarcomatous or rhabdomyosarcomatous [1-4]. The presence of rhabdomyoblastic elements is common in SC of other organs; however, it is exceedingly rare in the mucosa of the head and neck including the larynx [2,3]. Differentiating this entity from a mucosal-based sarcoma can be challenging, especially when the differentiated epithelial component is not identified [5]. We present a case of laryngeal SC with rhabdomyoblastic differentiation, misdiagnosed as a rhabdomyosarcoma (RMS) of the vocal cord.

## Case presentation

**Figure 1:**
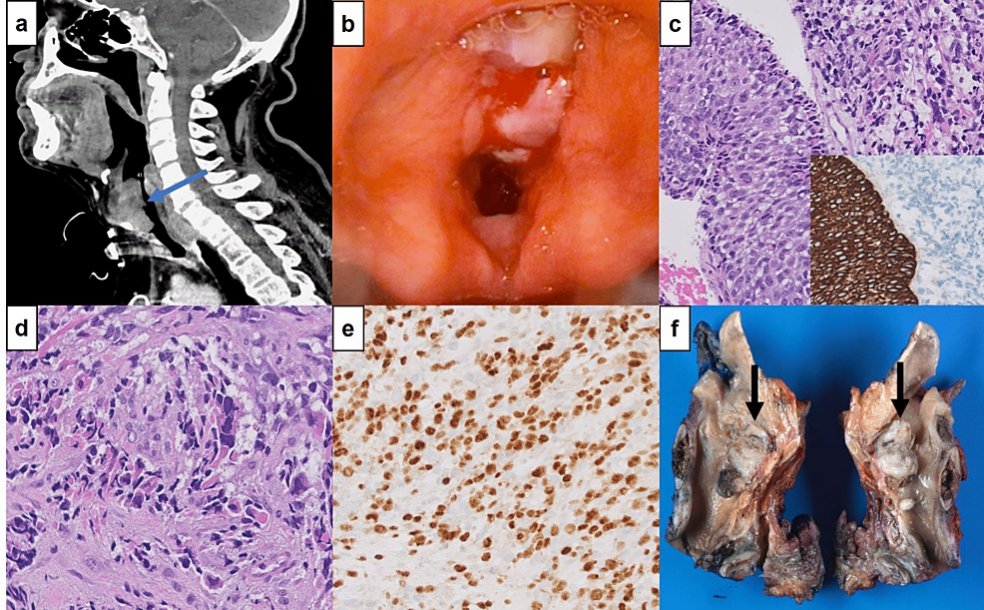
Radiologic and pathologic findings (1a) CT neck showing a 4.1 cm heterogeneously enhancing transglottic mass involving supraglottic, glottic, and subglottic regions of the larynx. 1(b) Laryngoscopy showing a large, polypoid mass, involving both left and right true vocal cords and false vocal cords. (1c) Carcinoma in situ adjacent to sarcomatous component (hematoxylin and eosin, x400); CK5/6 showing an immunopositivity in the squamous component but is negative in the sarcomatous component (inset, x200). (1d) High magnification of sarcomatous component showing spindle cells with eosinophilic cytoplasm and enlarged, eccentric, hyperchromatic nuclei with irregular nuclear borders indicative of rhabdomyoblastic differentiation (hematoxylin and eosin, x400). (1e) Myogenin showing diffuse nuclear staining in rhabdomyoblastic component (x200). (1f) Larynx, sagittal section, with large, exophytic glottic mass (arrows), 3.2 cm in greatest dimension.

A 72-year-old man with a past medical history of hypertension, hyperlipidemia, remote smoking history, and “changes in the voice” for the last 3 years was found unconscious. He was taken to the emergency room and stabilized after his arrest. He underwent a CT scan of the neck showing a large, heterogeneously enhancing transglottic mass involving supraglottis, glottis, and subglottic regions, measuring 4.1 cm in maximum dimension (Figure 1a). A CT of the chest, abdomen, and pelvis was negative for other masses or possible distant metastasis. Flexible laryngoscopy revealed a polypoid mass on bilateral true vocal cords and was followed by microlaryngoscopy with biopsy reported as pleomorphic RMS. The patient was transferred to our institution with this diagnosis for treatment.

Laryngeal biopsies of the mass were performed by direct laryngoscopy at our institution (Figure 1b), and microscopic examination showed a malignant neoplasm with biphasic morphology. The tumor showed a squamous component with high-grade dysplasia/carcinoma in situ (Figure 1c) and an invasive squamous cell carcinoma, diffusely admixing with a sarcomatous component consisting predominantly of malignant spindle-shaped cells and scattered large cells with eosinophilic cytoplasm, eccentric, hyperchromatic nuclei, and irregular nuclear borders suggestive of rhabdomyoblastic differentiation (Figure 1d), although no definitive skeletal muscle striations were identified. The immunohistochemical profile showed positive staining with AE1/AE3, CK5/6, and p40 in the epithelial component but negative in the sarcomatous component. The latter was strongly positive for desmin and myogenin (Figure 1e). Therefore, the diagnosis of SC with rhabdomyoblastic differentiation in this 72-year-old was made. The patient underwent a total laryngectomy with bilateral neck lymph node dissection. Grossly, the specimen revealed a large, lobulated, friable, mass measuring 3.2 cm in greatest dimension (Figure 1f) obliterating the right and left true vocal cords and extending into the subglottic region. Microscopic examination of the mass demonstrated the previously described findings, and no lymph node metastasis was identified. He is currently undergoing adjuvant radiation therapy.

## Discussion

SC accounts for about 2-4% of all malignancies in the larynx, the most common site being the glottis, followed by the supraglottic region [1-4]. This tumor has a peak incidence in the fifth and sixth decade of life with a mean age of 69 years. It is strongly associated with tobacco and alcohol abuse. Hoarseness is the most common symptom at presentation, and in our case, the malignancy was “incidentally” discovered while being treated for an episode of cardiac arrest, although after further inquiry, the patient referred change of voice for the last three years. SC, especially those of the larynx, are usually polypoid masses that are frequently ulcerated with associated necrosis [5]. Most commonly present at early stage T1, with very few cases presenting as T3 [4]. Microscopically, they can show a dysplastic squamous epithelium, carcinoma in situ, or invasive squamous cell carcinoma, but it is often inconspicuous and can be obliterated by the sarcomatous component [3]. The sarcomatous component usually exhibits a storiform, cartwheel, or whorled growth pattern of fusiform, rounded, or epithelioid cells. Heterologous mesenchymal elements like bone, cartilage, and rarely, skeletal muscle have been documented in 7-15% [2-5]. Immunohistochemical staining shows the spindle cells to be positive for p40, p63, and at least one cytokeratin in most cases [5-8]. However, cytokeratins and/or p63 and p40 reactivity may vary from focal to diffuse, or it can be negative. In up to 40% of cases, cytokeratin staining is negative [7], although the absence of cytokeratin and/or p63 and p40 staining does not exclude the diagnosis [5,9]. Vimentin is always strongly positive in the sarcomatous component, and desmin and actins may be present [3,5,7-10]. Rhabdomyoblastic differentiation can be highlighted by other myogenic markers including myogenin and myoglobin, helpful when the presence of cross striations in the rhabdomyoblasts is not clearly visualized [5]. In our case, both the carcinomatous and sarcomatous components were clearly identified on hematoxylin and eosin stains, and the immunohistochemical stains confirmed the rhabdomyoblastic features.

It has been documented that SC goes through an epithelial-mesenchymal transition, and this plasticity of interconversion is expressed by a loss of intercellular cohesion, elongation of the cells, and invasion of stroma [3,9,11,12]. This has been extensively studied by Zidar et al. [11] who found a loss of immunohistochemical expression of E-cadherin and N-cadherin, partial or complete loss of catenin expression, and an altered cadherin-catenin complex. Another study by the same author found down-regulation of the miR-200 family and miR-205 and loss of desmosomal cadherins which regulate epithelial-mesenchymal transition [12].

The main aspect of this case is this rare occurrence of rhabdomyoblastic features in laryngeal SC, which led to an initial misinterpretation of RMS. This type of differentiation in SC is uncommon in tumors of the mucosa in the head and neck region, and it is extremely rare in the larynx [2,3,13-16]. To the best of our knowledge, only three cases occurring in the larynx have been previously reported [14-16]. Srinivasan et al. [14] described the rhabdomyoblastic features as “scattered bizarre giant cells were seen. The most striking feature was the presence of striations, both transverse and longitudinal.” This case showed a squamous cell carcinoma component and was diagnosed as “carcinosarcoma,” a terminology that is not currently in use [1]. Doglion et al. [15] described a laryngeal carcinoma with rhabdomyoblastic features and positive desmin stain, but with an additional neuroendocrine component that is unique in this tumor. Goldman et al. [16] reported a patient case that presented with hoarseness and multiple lymph node metastases, which is also uncommon, as most patients present at an early stage [4].

As previously mentioned, this patient came from an outside institution with a diagnosis of laryngeal RMS. Primary sarcomas of the larynx are rare and represent 0.3-1% of all laryngeal malignancies [2]. The most common laryngeal sarcoma is chondrosarcoma. Primary RMS of the larynx is extremely rare, and when they occur, it tends to be in the pediatric population, as opposed to SC which has a peak incidence in the fifth to sixth decades of life [1,5]. In addition, these tumors tend to be located deeply seated at any location, whereas SC most commonly presents as an exophytic lesion protruding from a mucosal surface [5]. There are four forms of RMS: embryonal, alveolar, pleomorphic, and spindle/sclerosing [17]. Laryngeal RMS in adults tends to be an alveolar subtype, whereas pediatric ones are typically embryonal type [5]. Microscopically alveolar RMS shows fibrovascular septa separating cellular nests of small-sized monomorphic round cells with scant cytoplasm and tends to coalesce in the center with a discohesive periphery [16]. It harbors t(2;13) translocation which rearranges the PAX3 paired box gene, as well as the t(1;13) translocation which rearranges PAX7 on chromosome 1 and fuses to FKHR on chromosome 13 [18]. Embryonal RMS shows primitive round to spindle cells, with scant cytoplasm and hyperchromatic nuclei with scattered rhabdomyoblasts in a “cambium layer” [17]. Half of these tumors harbor RAS pathway mutation, MYOD1 mutation, loss of CDKN2A, amplification of FGFR4, and gain of GLI1 [13]. The spindle/sclerosing RMS has a fasciculated proliferation of spindle cells with elongated nuclei and pale indistinct cytoplasm, interspersed fusiform or polygonal rhabdomyoblasts [17]. Pleomorphic RMS is characterized by sheets of large, atypical, or bizarre, polygonal tumor cells [19]. This tumor occurs almost exclusively in adults and the pathogenesis remains unclear [19]. The morphologic features of this RMS subtype are more like our case, and this patient was referred to our institution for treatment with this diagnosis.

The WHO defines SC as composed of spindle/epithelioid pleomorphic cells usually associated with intraepithelial dysplasia and/or invasive squamous cell carcinoma [1]. If there is no morphologic evidence of an epithelial component, a demonstration of epithelial differentiation in the spindle cell component by immunohistochemistry can be helpful. However, up to 74% of SC are completely negative for epithelial markers [3,7,9,10,13]. The matter is further complicated when the spindle cell tumor lacks evidence of an epithelial component and expresses mesenchymal markers [5]. The immunoreactivity to myogenic markers in our case led to the misdiagnosis of RMS. However, when we reviewed the case, there were in situ and invasive squamous cell carcinoma components, which supported our final diagnosis of SC with rhabdomyoblastic differentiation. Given the extreme rarity of SC with rhabdomyoblastic features in the larynx of middle-aged adults and the elderly, and spindle cell neoplasm with exophytic appearance located in the superficial submucosa should be considered SC, even when no morphological or immunohistochemical evidence of a malignant epithelial component [5]. The 2022 WHO Classification of Tumors in its Head and Neck chapter states “a mucosa-based malignant spindle cells neoplasm is a spindle cell squamous carcinoma until proven otherwise” [1].

## Conclusions

SC with rhabdomyoblastic features is extremely rare in the larynx, and differentiating it from sarcomas is challenging, especially in cases where epithelial differentiation is not evident. Pathologists should be aware of the possibility of finding a rhabdomyoblastic heterologous component in SC arising in the larynx, and they should consider this diagnosis, especially in an older patient with a superficially situated exophytic tumor, even when evidence of epithelial differentiation is lacking, before committing to a diagnosis of RMS.
